# Modified TPP-MoS_2_ QD Blend as a Bio-Functional Model for Normalizing Microglial Dysfunction in Alzheimer’s Disease

**DOI:** 10.3390/neurolint15030061

**Published:** 2023-08-08

**Authors:** Ohoud A. Alomari, Safaa Qusti, Maha Balgoon, Fadwa Aljoud, Khalid A. Alamry, Mahmoud A. Hussein

**Affiliations:** 1 Department of Biochemistry, Faculty of Science, King Abdulaziz University, Jeddah 21589, Saudi Arabia; 2 Regenerative Medicine Unit, King Fahd Medical Research Centre, King Abdulaziz University, Jeddah 21589, Saudi Arabia; 3 Department of Chemistry, Faculty of Science, King Abdulaziz University, Jeddah 21589, Saudi Arabia; 4 Chemistry Department, Faculty of Science, Assiut University, Assiut 71516, Egypt

**Keywords:** Alzheimer’s disease, microglial dysfunction, amyloid beta (Aβ), modified TPP MoS_2_ QDs

## Abstract

Alzheimer’s disease (AD) is the most prevalent neurodegenerative disease of old age. Accumulation of β-amyloid peptide (Aβ) and mitochondrial dysfunction results in chronic microglial activation, which enhances neuroinflammation and promotes neurodegeneration. Microglia are resident macrophages of the brain and spinal cord which play an important role in maintaining brain homeostasis through a variety of phenotypes, including the pro-inflammatory phenotype and anti-inflammatory phenotypes. However, persistently activated microglial cells generate reactive species and neurotoxic mediators. Therefore, inhibitors of microglial activation are seen to have promise in AD control. The modified TPP/MoS_2_ QD blend is a mitochondrion-targeted nanomaterial that exhibits cytoprotective activities and antioxidant properties through scavenging free radicals. In the present study, the cell viability and cytotoxicity of the DSPE-PEG-TPP/MoS_2_ QD blend on microglial cells stimulated by Aβ were investigated. The levels of reactive oxygen species (ROS) and mitochondrial membrane potential (MMP) were also assessed. In addition, pro-inflammatory and anti-inflammatory cytokines, such as tumor necrosis factor α (TNF-α), interleukin-6 (IL-6), interleukin-1β (IL-1β), transforming growth factor beta (TGF-β), inducible nitric oxide synthase (iNOS) and arginase-1 (Arg-I) were measured in the presence or absence of the DSPE-PEG-TPP/MoS_2_ QD blend on an immortalized microglia cells activated by accumulation of Aβ. We found that the DSPE-PEG-TPP/MoS_2_ QD blend was biocompatible and nontoxic at specific concentrations. Furthermore, the modified TPP/MoS_2_ QD blend significantly reduced the release of free radicals and improved the mitochondrial function through the upregulation of MMP in a dose-dependent manner on microglial cells treated with Aβ. In addition, pre-treatment of microglia with the DSPE-PEG-TPP/MoS_2_ QD blend at concentrations of 25 and 50 μg/mL prior to Aβ stimulation significantly inhibited the release and expression of pro-inflammatory cytokines, such as IL-1β, IL-6, TNF-α, and iNOS. Nevertheless, the anti-inflammatory cytokines TGF-β and Arg-I were activated. These findings suggest that the modified TPP/MoS_2_ QD blend reduced oxidative stress, inflammation and improved the mitochondrial function in the immortalized microglial cells (IMG) activated by Aβ. Overall, our research shows that the DSPE-PEG-TPP/MoS_2_ QD blend has therapeutic promise for managing AD and can impact microglia polarization.

## 1. Introduction 

Alzheimer’s disease (AD) is the most prevalent form of dementia. It is characterized by extracellular amyloid plaque deposition and intracellular neurofibrillary tangles, which lead to cognitive failure [1,2]. Both mitochondrial dysfunction and the accumulation of Aβ aggregates in the AD brain cause neuroinflammation and microglial activation, which eventually result in neuronal death and brain atrophy [3,4,5]. Microglia are specific immune cells in the central nervous system that act as macrophages in the brain and regulate tissue homeostasis through a variety of phenotypes [6], including, but not limited to the pro-inflammatory and anti-inflammatory phenotypes. The harmful phenotypes are primarily mediated by redox signaling and the activation of proinflammatory mediators, while the protective phenotypes are involved in neural defense mechanisms such as Aβ clearance, anti-inflammation, and antioxidant pathways [6,7]. However, the overall response of these cells is complex and dependent on the diseased circumstances and inflammation state [8]. Sustained microglial activation induced by Aβ contributes greatly to the pathogenic processes in AD through the continued release of potentially cytotoxic molecules, such as proinflammatory cytokines and reactive oxygen intermediates [9,10]. Additionally, disorders of mitochondrial energy metabolism and metabolic reprogramming can result in chronically activated microglia [11]. Therefore, adverse regulators of microglial activation are now considered potential therapeutic candidates for AD [12,13].

Molybdenum disulfide (MoS_2_) nanomaterials have been widely used in biomedical research because of their unique physical and chemical properties [14]. When MOS_2_ structure is scaled down from bulk to a very thin layer of monolayer quantum dots (QDs), it exhibits unique and interesting catalytic activities which enable MoS_2_ to be considered as a promising nanoplatform for various medical applications in diagnosis and disease therapies [15,16]. MoS_2_ at the nano scale can work as an excellent antioxidant, scavenging different types of free radicals via nanozyme activity, which mimics the intrinsic major cellular antioxidant enzymes [17,18,19]. In addition, previous research has demonstrated that MoS_2_ NPs have multifunctional effects on the AD model by significantly reducing cytotoxicity and Aβ aggregation [20,21]. Furthermore, 1,2-Distearoyl-sn-glycero-3-phosphoethanolamine-Poly (ethylene glycol) conjugated to 3-Carboxypropyl triphenyl-phosphonium bromide (DSPE-PEG-TPP) is a polymer that is used in drug administration applications and helps to enhance the stability and circulation time of the medication. Moreover, it can target mitochondria by attracting their highly negative membrane charge [22].

Studies on the use of modified DSPE-PEG-TPP-MoS_2_ QDs in the management of diseases are extremely limited. But a previous study showed that modified TPP-MoS_2_ QDs are important for reducing the development of Alzheimer’s disease and the polarization of microglia [23]. However, it is important to note that the results were achieved with different cell lines and experimental setups. This investigation used IMG obtained from adult C57BL/6J mouse brains at 8 weeks of age. Proliferation and retroviral infection were carried out on a growth medium specifically designed to promote the development of these cells.

The aim of this work was to highlight the beneficial role of the DSPE-PEG TPP/MoS2 QD blend for normalizing IMG dysfunction mediated by Aβ in the Alzheimer’s disease model.

## 2. Materials and Methods

### 2.1. Materials

The immortalized microglial cells (IMG) cell line was purchased from Kerafast (product number EF4001, Boston, MA, USA). Dulbecco’s Modified Eagle’s Medium (DMEM), fetal bovine serum (FBS), penicillin-streptomycin solution, trypsin-EDTA solution, phosphate-buffered saline (PBS), Thiazolyl Blue Tetrazolium Bromide powder (MTT), dimethyl sulfoxide (DMSO), DAPI stain, chloroform, 111,333-hexafluoro-2-propanol (HFIP) and lyophilized amyloid beta 1–42 peptide (Aβ) were all supplied by Sigma–Aldrich Chemical Corporation (St. Louis, MO, USA). F4/80(11-4801-81) and CD86(12-0862-81) were provided by Thermo Fisher Scientific (Waltham, MA, USA). Molybdenum disulfide nanoparticles (MoS2 QDs) and DSPE-PEG-TPP were obtained from XFNANO (product number XF186, Nanjing, Jiangsu, China) and Biopharma PEG Scientific Inc. (product number LP096175, Watertown, MA, USA), respectively.

### 2.2. Preparation of Modified TPP-MoS_2_ Blend

MoS_2_ QDs (10 mg) and DSPE-PEG-TPP (25 mg) were blended and mixed in 25 mL of chloroform. Next, the mixture was evaporated and dried using a lyophilizer for 24 h. Then, 10 mg of the produced powder was weighed and resuspended in de-ionized water (10 mL) as a stock solution with a final concentration of 1000 μg/mL. Sonication was then performed for 2 h. Free debris was removed by dialysis using dialysis tubing with a 10 kDa MWCO for 24 h. Subsequently, the resultant solution was filtered through a 0.22 μM microporous membrane.

A preliminary study using a wide range of concentrations (1000–1 μg/mL) was conducted, and it was determined that the optimal concentration for cell survival for 72 h was less than 100 μg/mL. Subsequently, two-fold serial dilutions starting from 100 μg/mL were applied on treated cells using culture media as diluent. 

Transmission electron microscopy (TEM, Hitachi HT7700, Hitachi, Honshu, Japan) was used to measure the diameters of modified TPP-MoS_2_ NPs.

### 2.3. Preparation of Aβ Solution

The lyophilized amyloid peptide was dissolved in HFIP to generate 1 mM A Monomer. The sample was vortexed and centrifuged to produce a clear solution, which was then aliquoted into small Eppendorf tubes and placed in a fume hood overnight to allow the HFIP to completely evaporate before being stored at −20 °C. The acquired samples were dissolved in DMSO, vortexed, bath sonicated, centrifuged, and diluted to 100 μM with cold PBS + 0.05% SDS in order to create oligomeric Aβ. The Aβ aggregates were incubated at 4 °C to promote high-order aggregation [23,24]. Before treating the cells, the oligomeric Aβ was further diluted to 20 μM in culture medium. SDS-PAGE Gel Electrophoresis was performed to characterize Aβ Aggregates.

### 2.4. Cell Culture and Treatment

IMG cells were cultured in Dulbecco’s Modified Eagle’s Medium containing 10% fetal bovine serum and 1% penicillin-streptomycin at 37 °C with 5% CO_2_ and 95% O_2_. Trypsinization with 0.25% trypsin was used to separate adhering cells from confluent IMG cultures. Flow cytometry was used to analyze the specific microglia markers F4/80 and CD86 (FACSCalibur, BDBiosciences, San Diego, CA, USA).

Then, varying concentrations of DSPE-PEG-TPP/MoS_2_ QD blend were utilized to pre-treat IMG cells, while 20 μM of Aβ were employed to co-treat IMG cells for triggering microglial activation. 

### 2.5. Cell Viability Assay and Morphological Studies 

Cell viability was determined using the colorimetric MTT assay, which measures cell mitochondrial metabolic activity. IMG microglia cells were seeded in a 96-well plate at a density of 4000 cells in 100 μL of cell culture medium per well. Cells were incubated overnight at 37 °C with 5% CO_2_. The next day, cells were pre-treated with several concentrations of DSPE-PEG-TPP/MoS_2_ QD blend (6.25–50 μg/mL) for 8 h prior to the addition of 20 μM of Aβ, in line with previous experimental practice [24]. After 24 h of incubation, old media was removed, and 0.5 mg/mL of MTT solution was added to each well and incubated at 37 °C for 4 h. Media were aspirated, and formazan crystals were dissolved in 100 μL of DMSO, and the optical density (OD) was measured at 570 nm using a microplate reader (BioTek ELX808, Winooski, VT, USA). Cell viability was expressed as a relative measure, with the control representing 100% cell viability.

Following the selection of the optimal concentrations of modified TPP/MoS2 QD blend based on the MTT experiment, the cell morphology was examined using a light microscope (TH4-200, Olympus optical Co-Ltd., Tokyo, Japan).

### 2.6. Morphological Assessment of Apoptotic Cells Nuclei with DAPI Staining

DAPI staining was used to detect DNA fragmentation and nuclear abnormalities in treated cells. Cells were seeded into the 96-well plates at a density of 4 × 103 cells/well. The cells were then pre-treated for 8 h with different (optimal) concentrations (6.26–50 μg/mL) chosen based on the MTT test results. Cells were then exposed to Aβ (20 μM) for 16 h. After 24 h of incubation, the morphology of the DAPI-stained cells was evaluated using a fluorescence microscope with a blue filter at 437 m (Leica CRT6000, Wetzlar, Germany) and quantification of apoptotic percentage according to [25].

### 2.7. Intracellular Reactive Oxygen Species (ROS) Level

The level of intracellular ROS was measured using a fluorescent product formed by the oxidation of dichlorofluorescein (DCF). IMG microglial cells were plated into 96-well plates at a density of 2 × 104 cells/well overnight. Cells were then treated with DSPE-PEG-TPP/MoS_2_ QD blend (6.26–50 μg/mL) for 8 h and then exposed to Aβ (20 μM) for 16 h. After 24 h, 10 μM DCFH-DA solution was added directly to cells and incubated for 30 min in the dark. The cells were then washed to remove excess DCFH-DA that had not entered the cells. The fluorescence intensity of the oxidized product in culture media was measured using a fluorescence microscope and a fluorescence spectrophotometer at 488/525 nm (BioTek ELX800, Winooski, VT, USA). Values were expressed as the percentage of fluorescence intensity relative to the control wells.

### 2.8. Mitochondrial Membrane Potential (MPP) Assay

Changes in mitochondrial membrane potential were assessed using a JC-10 assay kit according to the manufacturer’s protocol (Solarbio Life Science Company, Beijing, China). Cells were plated overnight at 5 × 10^4^ cells/well in a 96-well plate and then treated with DSPE-PEG-TPP/MoS_2_ QD blend (6.26–50 μg/mL) for 8 h, followed by 20 μM of Aβ for 16 h. Working solutions of JC-10 dye were then added to the wells and incubated for 30 min (37 °C); then, the assay buffer was added. The fluorescence intensities for both JC-10 monomeric and aggregates forms were measured at Ex/Em = 485/515 nm and Ex/Em = 540/590 nm using a fluorescence microscope and microplate reader (BioTek ELX800, Winooski, VT, USA). Change in mitochondrial membrane potential was expressed as the ratio between the aggregate and monomer.

### 2.9. Cytokine Assay

(a) Enzyme-Linked Immunosorbent Assay (ELISA)

IMG microglial cells were pre-treated with DSPE-PEG-TPP/MoS_2_ QD blend (25 and 50 μg/mL) for 8 h, then exposed to Aβ (20 μM) for 16 h. After 24 h, culture media from the cells were collected, and concentrations of IL-1β, IL-6, TNF-α, and TGF-β were measured using ELISA kits in accordance with the manufacturer’s instructions (Solarbio Life Science Company, Beijing, China). The OD was measured at 450 nm using an ELX808 microplate reader.

(b) Quantitative Polymerase Chain Reaction

Gene expression at the mRNA level was measured by real-time PCR analysis. Briefly, total RNA was extracted from treated IMG microglial cells using RNeasy kit (Qiagen, Germantown, MD, USA) according to the manufacturer’s protocols. An ImProm-II Reverse Transcription System (Promega, CA, USA) was used to synthesize cDNA from 1 μg of isolated RNA. Prepared cDNA was used for the quantitative polymerase chain reaction (qPCR) analysis using 2× Taq PCR Master Mix reagent BioFact™ (Daejeon, Republic of Korea) and gene-specific primers. Relative mRNA levels were estimated by comparative analysis of Ct values (ΔΔCt method) and normalized to values measured for glyceraldehyde 3-phosphate dehydrogenase (GAPDH) within the same samples. Table 1 shows the primer sequences used in this study.

### 2.10. Statistical Analysis of Data

Data analysis was performed using GraphPad Prism 8 software (GraphPad Software Inc., San Diego, CA, USA). Data were expressed as the mean ± standard deviation from at least three separate experiments. Differences between groups were analyzed using one-way analysis of variance (ANOVA, New Providence, NJ, USA) followed by Tukey’s multiple comparison test. A value of *p* < 0.05 was considered statistically significant.

## 3. Results and Discussion

There are no effective treatments for Alzheimer’s disease, which is becoming more common among the aged population. Unfortunately, current medications only reduce symptoms for a limited time; they cannot stop or prevent the disease from progressing. As a result, there is an urgent need to develop an effective treatment agent [26]. Numerous studies have concluded that microglial activation is linked with the progression of AD and that preventing such activation can help to regulate the neurodegenerative process [27]. Developing a nano-formulation treatment that can cross the blood–brain barrier (BBB) and has highly specific targeting properties to regulate microglial activity may be considered a therapeutic strategy for the treatment of AD [28]. In this study, we investigated the effect of a DSPE-PEG-TPP/MoS_2_ QD blend on IMG cells activated by Aβ aggregate.

**Figure 1:**
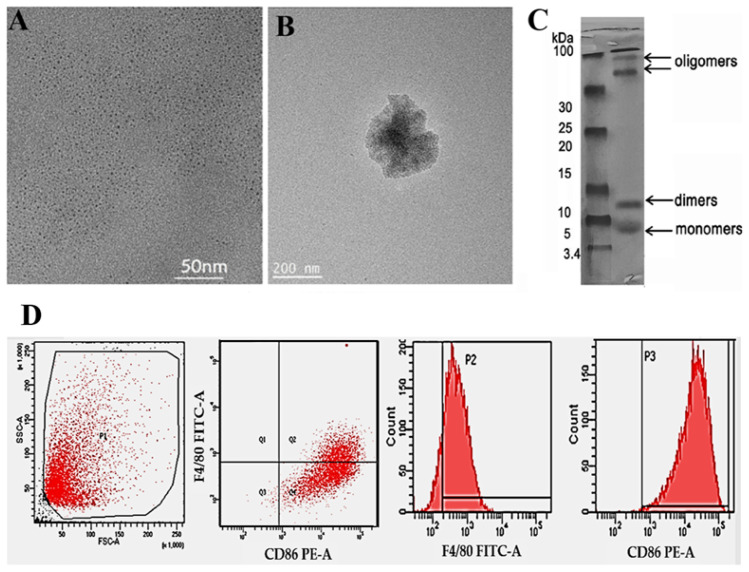
TEM images showing the diameters of (**A**) pure MoS_2_ QDs; (**B**) modified DSPE-PEG-TPP/MoS_2_ QDs; (**C**) Aβ Aggregate was detected using SDS-PAGE gel; (**D**) certain microglia markers are identified using flow cytometry.

First, TEM measurements were used to learn about the DSPE-PEG-TPP/MoS_2_ QD blend. These results were compared to those for pure MoS_2_ QDs, as shown in Figure 1A,B. The nanocomposite size was around 100 nm, which is acceptable for crossing the BBB as small medicine appears to have more therapeutic efficacy in the treatment of AD [28]. Moreover, the SDS-PAGE Gel was performed to characterize the Aβ solution and ensure the toxic aggregation produced in Figure 1C. Additionally, the primary microglia markers F4/80 and CD86 expression allowed for the identification of IMG cells. Moreover, the microglia-specific marker CD86 is significantly expressed by activated microglia. Our findings confirm that both markers were stained in more than 89% of cells after treatment with Aβ Figure 1D.

**Figure 2:**
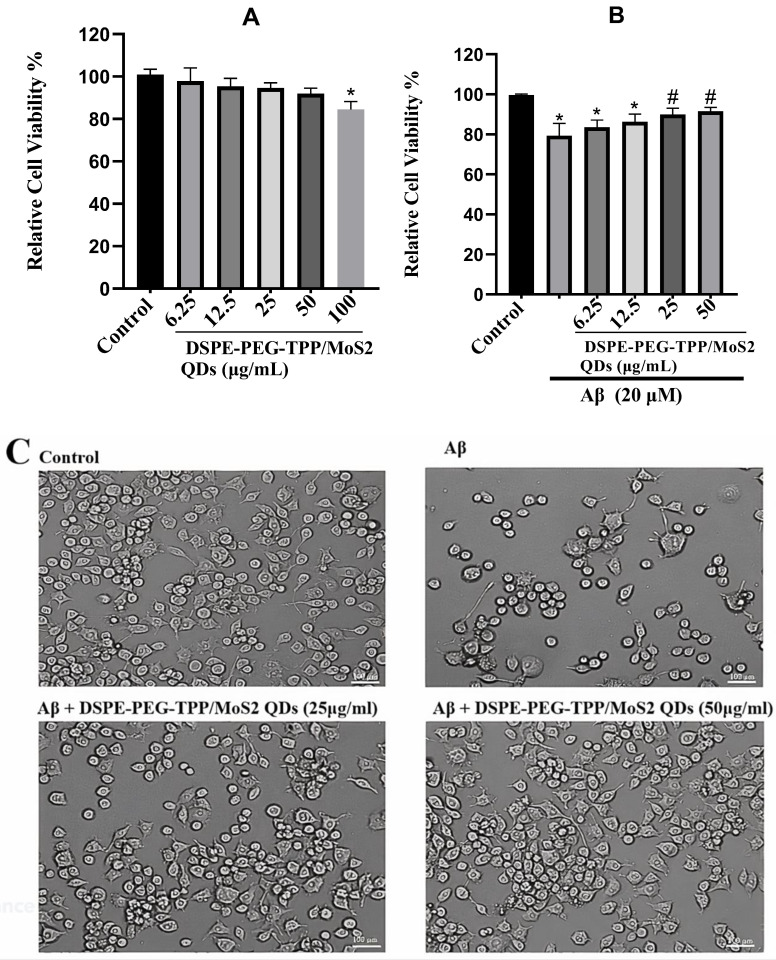
(**A**) Cell viability of the DSPE-PEG-TPP/MoS_2_ QD blend on the microglial cell line was determined using MTT assay. (**B**) Protection effects of DSPE-PEG-TPP/MoS_2_ QD blend on Aβ-mediated cytotoxicity of IMG cells using the MTT assay. (**C**) Microscopy demonstrating the effect of DSPE-PEG-TPP/MoS_2_ QD blend on Aβ-induced cytotoxicity in IMG cells. Scale bar, 100 µm. * *p* < 0.05 compared to the control group. # *p* < 0.05, compared to the Aβ group.

Cell viability was then measured using an MTT assay. The results showed that cells maintained a high viability of more than 90%, demonstrating that the DSPE-PEG-TPP/MoS_2_ QD blend was biocompatible and safe at certain concentrations. In contrast to treatment with Aβ, around a quarter of the cells died following treatment with 20 μM. Furthermore, the combination of DSPE-PEG-TPP/MoS_2_ QD blend with Aβ greatly reduced the cytotoxicity of Aβ and significantly increased cellular protection in a dose-dependent manner (Figure 2A,B). The cell viability data showed that about 89.9 and 91.5% of cells were alive after combinations of 25 and 50 μg/mL of DSPE-PEG-TPP/MoS_2_ QD blend, with the amyloid peptides, respectively. This finding is consistent with those of earlier studies which found that MoS_2_ nanomaterials have very good biocompatibility, low toxicity, high stability, and acceptable dispersion in biological systems [29]. MoS_2_ is composed of elements (molybdenum and sulfur) that exist in the human body. Molybdenum is a trace dietary element that plays a key role in human metabolism by catalyzing redox and oxygen-transfer reactions. In addition, sulfur has an important role in the formation and activation of different cellular proteins [30]. However, higher concentrations of the blood can induce cell toxicity as concentrations above 100 μg/mL reduce the cells’ viability. Depending on their physicochemical characteristics, MoS_2_ nanoparticles can be harmful to biological systems [31]. However, more research on the toxicity of this blend should be conducted.

Cell morphologies were evaluated using a light microscope for IMG cells. As can be seen in the image in Figure 2C, control (untreated) microglia cells appeared healthy and exhibited processes and a ramified morphology which contrasted with Aβ-stimulated cells, whose morphology turned amoeboid during microglial activation. Cells treated with DSPE-PEG-TPP/MoS_2_ QD blend prior to Aβ addition were similar to controls with minor differences that indicated the blend retained and preserved the morphology of microglia exposed to Aβ.

**Figure 3:**
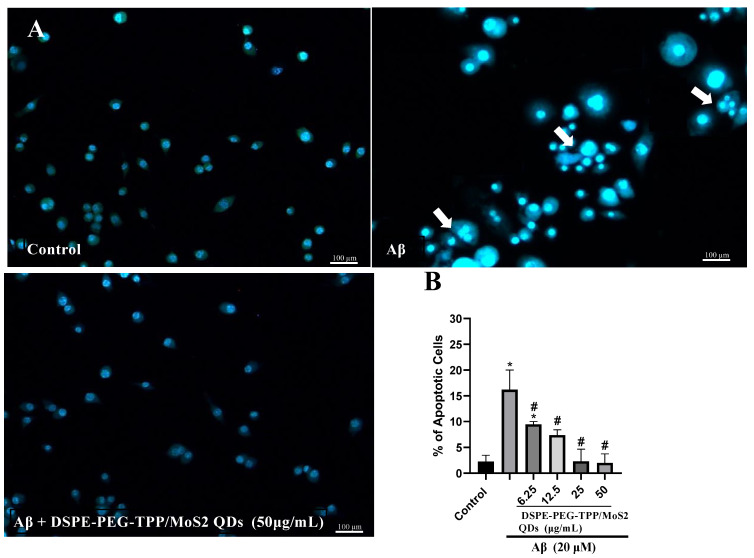
The DSPE-PEG-TPP/MoS2 QD blend decreases Aβ-induced apoptosis in IMG cells stained with DAPI. (**A**) Observed using fluorescence microscopy, with exhibited apoptotic changes highlighted by arrows. (**B**) Quantification data of apoptotic cells. Scale bar, 100 µm. * *p* < 0.05 compared to the control group. # *p* < 0.05, compared to the Aβ group.

Subsequently, a DAPI stain was applied to evaluate changes in nuclear morphology after the treatment of IMG cells. Under the fluorescent microscope, the nuclei of healthy control cells were large and showed smooth, uniform, and diffused staining; however, the nuclei of Aβ displayed abnormalities in nuclear morphology. This clearly illustrated an early activation of apoptosis. Aβ caused nucleus enlargement and a decrease in cell population; however, when cells were treated with Aβ in combination with the DSPE-PEG-TPP/MoS_2_ QD blend, the apoptotic characteristics were reduced (Figure 3). Our findings were consistent with those of a prior investigation that found Aβ induced apoptosis, which can be regulated by pretreatment with Acrp30 (a globular analogy of adiponectin) in Aβ-exposed BV2 microglia [32]. 

**Figure 4:**
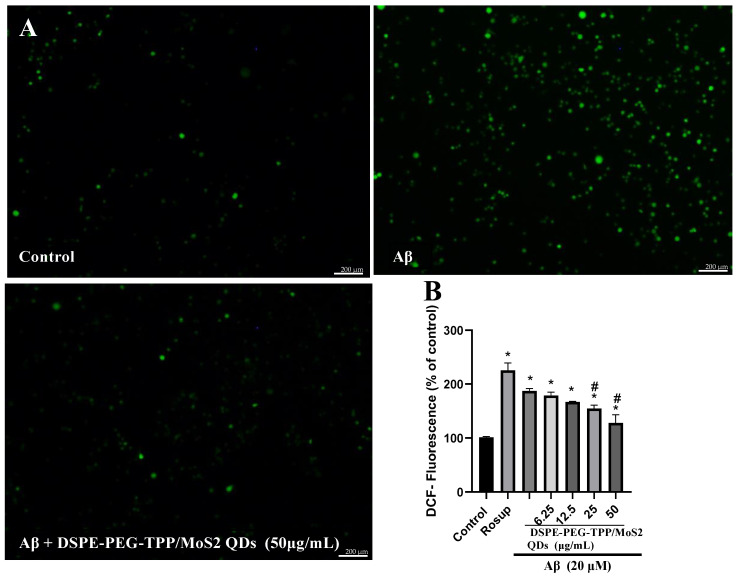
The DSPE-PEG-TPP/MoS_2_ QD blend protects IMG cells from Aβ-induced oxidative stress, as determined by the DCFH-DA assay. (**A**) Visualization using fluorescence microscopy. (**B**) Detection using a fluorescence microplate reader. Scale bar, 200 µm. * *p* < 0.05 compared to the control group. # *p* < 0.05, compared to the Aβ group. DCF, dichlorodihydrofluorescein diacetate; Rosup, positive control.

Next, the intracellular ROS level was determined. In IMG cells, strong fluorescence intensity triggered by Aβ was obvious, indicating that a high level of ROS was produced. In comparison to the untreated control cells, Aβ significantly increased the generation of intracellular ROS to 89.6%. The combination of the DSPE-PEG-TPP/MoS_2_ QD blend with Aβ significantly reduced the intracellular ROS level in a dose-dependent manner (Figure 4). The excessive production of ROS causes oxidative stress, which is a key phenomenon in AD. Numerous studies have concluded that antioxidants are a powerful therapeutic option that can neutralize free radicals and prevent neuroinflammation by improving the protective effect of microglia [33]. However, although traditional antioxidants are used widely in different neurodegenerative diseases, they are still not very effective due to their poor pharmacokinetic and pharmacodynamic properties. The use of nanoparticles may overcome these limitations and lead to considerable improvements in the pharmacological profiles of therapeutic molecules [34]. In addition, nanomaterials have demonstrated a persistent effect and a directed delivery system. MoS_2_-based nanomaterials can work as nanozymes, and these have been shown to exhibit antioxidant properties with superoxide dismutase (SOD) and catalase (CAT) mimicking activity, which contributes to downregulating free radicals and inflammation, according to the results of in vivo and in vitro experiments [35]. Additionally, MoS_2_ nanoparticles have been shown to significantly reduce oxidative stress and amyloid pathology in an Alzheimer’s model [36].

**Figure 5:**
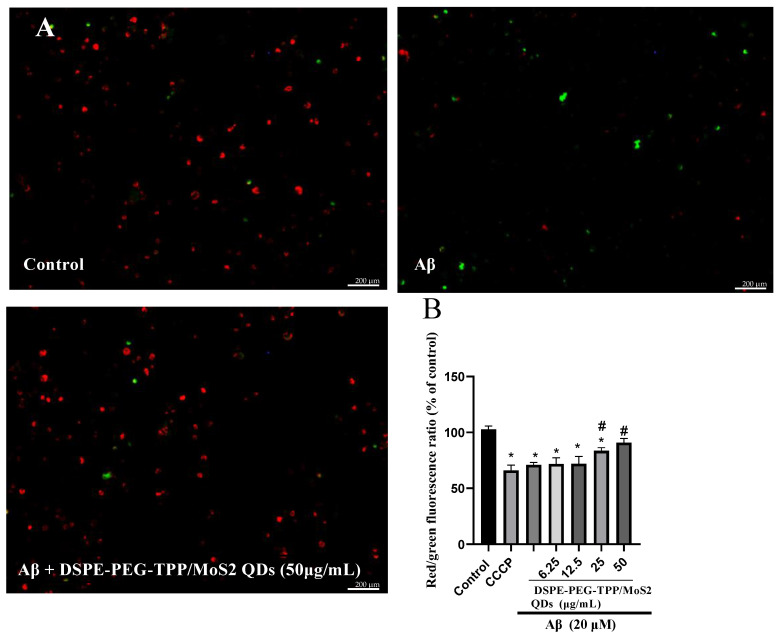
The DSPE-PEG-TPP/MoS_2_ QD blend protects IMG cells from Aβ-induced mitochondrial dysfunction, as determined by changes in mitochondrial membrane potential identified with JC-10 dye. (**A**) Visualization using fluorescence microscopy. (**B**) Detection using a fluorescence microplate reader. Scale bar, 200 µm. * *p* < 0.05 compared to the control group. # *p* < 0.05, compared to the Aβ group. JC-10, 5,5′,6,6′-tetrachloro-1,1′,3,3′-tetraethylbenzimidazol-carbocyanine iodide; Carbonyl cyanide 3-chlorophenyl-hydrazone (CCCP) positive control.

Following that, changes in the mitochondrial membrane potential (MMP) of microglia cells were examined after the cells were stained with JC-10, a cationic dye that is able to selectively accumulate in mitochondria and which reversibly changes color from green to red as membrane potential increases. When compared to the control, microglia activated by Aβ were found to exhibit a significantly lower MMP and greater mitochondrial depolarization determined by the aggregate/monomer (red/green) fluorescence intensity ratio. However, pre-treatment with a high concentration of DSPE-PEG-TPP/MoS_2_ QD blend significantly reduced the amount of green fluorescence, increased the amount of red fluorescence, and normalized Aβ-induced mitochondrial damage (Figure 5). Mitochondria are highly active organelles responsible for ATP generation as well as basic cellular activities, such as cell survival and death, redox state, and responding to external stimuli. Disturbances in the balance of mitochondrial dynamics, as evidenced by a shift toward fission, have been linked to a range of illnesses, including AD [37]. A growing body of evidence indicates that microglial activation and neuroinflammation are linked to mitochondrial dysfunction. Accumulation of Aβ in AD patients’ brains causes damage to mitochondrial DNA, alters mitochondrial metabolism, and disturbs MMP [38]. Our findings show that Aβ significantly simulates mitochondrial failure via depolarization of mitochondrial MMP; otherwise, pre-treatment with DSPE-PEG-TPP/MoS_2_ QD blend can prevent MMP decreases.

One previous study found that flower-like MoS_2_ nanosheets (FL-MoS_2_) caused endothelial cells to shift toward mitochondrial fusion rather than fission, as demonstrated by increased expression of mitochondrial fusion proteins (Mfn2, Opa1) and decreased expression of fission proteins (Fis1) which promote mitochondrial homeostasis [39].

**Figure 6:**
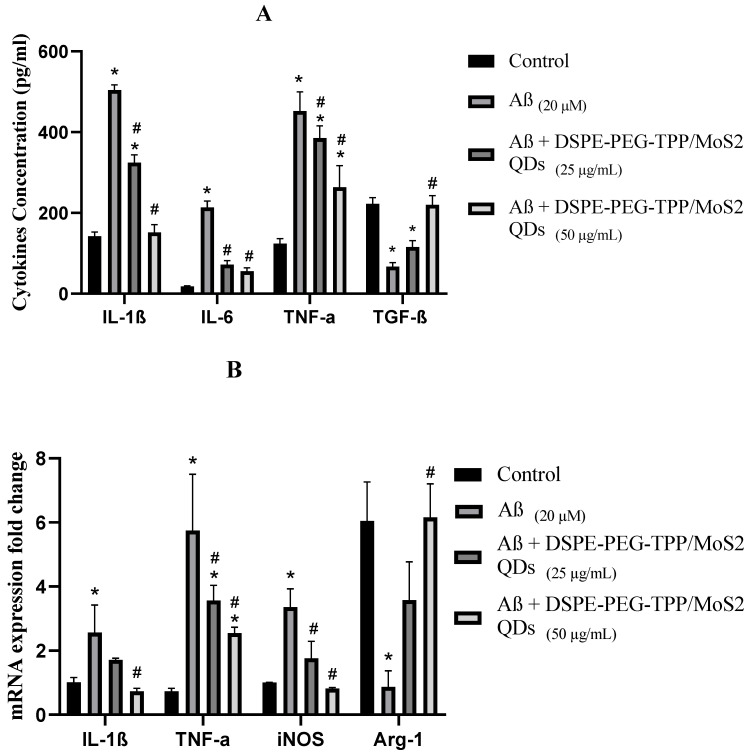
The DSPE-PEG-TPP/MoS_2_ QD blend protects IMG cells from Aβ-induced inflammation. (**A**) Levels of proinflammatory cytokines secreted by microglia cells determined by ELISA. (**B**) Gene expression of pro-inflammatory cytokines in microglia cells determined by real-time PCR analysis. * *p* < 0.05 compared to the control group. # *p* < 0.05, compared to the Aβ group.

Lastly, the anti-inflammatory impact of the DSPE-PEG-TPP/MoS_2_ QD blend on microglial cells activated by Aβ was determined using the ELISA technique and real-time PCR analysis. The results indicated that Aβ treatment significantly increased the levels of TNF-α, IL-1β, IL-6, and iNOS, which were partially suppressed by pre-treatment with the DSPE-PEG-TPP/MoS_2_ QD blend in a dose-dependent manner. Additionally, Aβ treatment significantly decreased the levels of TGF-β and Arg-1, both of which were elevated by pre-treatment with the DSPE-PEG-TPP/MoS_2_ QD blend. As can be seen from the data, the DSPE-PEG-TPP/MoS_2_ blend regulates microglial activation by reducing the cytokine level of inflammatory markers and increasing the level of cytoprotective markers (Figure 6).

Abundant studies have demonstrated the anti-inflammatory action of MoS_2_ nanoparticles on different cells [39,40], and the results of that study support our findings. The mechanisms of action by which the DSPE-PEG-TPP/MoS_2_ QD blend was able to produce neuroprotective effects and reduce inflammation were not mentioned; however, many studies using natural products and pharmacological drugs have reported that a protective effect against Aβ oligomers-induced microglial activation and inflammation cascades is achieved via suppression of the master switch for microglial activation, i.e., the transcription factor nuclear factor kappa-light-chain-enhancer of activated B cells (NF-κB), which plays a crucial regulatory function in the generation of inflammatory mediators resulting in neurotoxicity. On the other hand, the neuroprotective effect of activating nuclear factor E2-related factor 2 (Nrf2), which promotes the expression of cytoprotective and antioxidant genes, has been shown in previous research [41].

## 4. Conclusions

The modified TPP/MoS2 QD blend has cytoprotective and antioxidant properties because it can get rid of free radicals. This makes it an ideal nanomaterial for targeting mitochondria. This study investigated the effect of a DSPE-PEG-TPP/MoS_2_ QD blend on an IMG cell line activated by Aβ. Furthermore, the cytotoxicity of the DSPE-PEG-TPP/MoS_2_ QD blend on microglial cells stimulated by Aβ was investigated, together with the levels of reactive oxygen species (ROS) and mitochondrial membrane potential (MMP). The production and manifestation of pro-inflammatory cytokines were dramatically suppressed after microglia were pre-treated with the DSPE-PEG-TPP/MoS_2_ QD combination at doses of 25 and 50 g/mL prior to Aβ encouragement. The results additionally revealed that, through numerous bifunctional effects, a MoS_2_-based blend could effectively inhibit Aβ triggered microglia activation via antioxidant and anti-inflammatory capabilities. 

## Figures and Tables

**Table 1:** The primer (Eurofins Genomics, USA) sequences used were as follows:

	Forward	Reverse
GAPDH	ACTCCACTCACGGCAAATTC	TCTCCATGGTGGTGAAGACA
TNF-α	AAATGGCCTCCCTCTCATCAG	GTCACTCGAATTTTGAGAAGATGATC
IL-1β	AGCTTCAGGCAGGCAGTATC	AAGGTCCACGGGAAAGACAC
iNOS	GTTCTCAGCCCAACAATACAAGA	GTGGACGGGTCGATGTCAC
Arg-1	CACAGTCTGGCAGTTGGAAG	GGGAGTGTTGATGTCAGTGTG

## Data Availability

The data presented in this study are available on request from the corresponding author.
